# Angiogenesis in Non-Hodgkin's Lymphoma: An Intercategory Comparison of Microvessel Density

**DOI:** 10.5402/2012/943089

**Published:** 2012-03-27

**Authors:** Deepti Aggarwal, Gunjan Srivastava, Ruchika Gupta, Leela Pant, Gopal Krishan, Sompal Singh

**Affiliations:** ^1^Department of Pathology, Hindu Rao Hospital, Malka Ganj, Delhi 110007, India; ^2^Department of Pathology, Chacha Nehru Bal Chikitsalaya, Geeta Colony, Delhi 110031, India; ^3^Department of Medicine, Hindu Rao Hospital, Malka Ganj, Delhi 110007, India

## Abstract

*Background*. This study was aimed at comparing angiogenesis, seen as microvessel density (MVD) in subtypes of non-Hodgkin's lymphoma (NHL). *Methods*. In this study, 64 cases of NHL diagnosed over a three-year period were included along with 15 lymph node biopsies of reactive hyperplasia. NHLs were classified using REAL classification, and immunohistochemistry was performed for CD34 in all cases. CD34-stained sections were evaluated for “hot spots,” where MVD was assessed and expressed as per mm^2^. Appropriate statistical methods were applied. *Results*. There were 6 cases of well-differentiated lymphocytic lymphoma (SLL), 21 diffuse large B-cell lymphoma (DLBCL), 15 follicular lymphoma, 10 lymphoblastic lymphoma, 7 MALToma, and 5 peripheral T-cell lymphoma (PTCL). Mean MVD was highest in reactive hyperplasia (191.92 ± 12.16 per mm^2^) compared to all NHLs. Among NHLs, PTCL demonstrated the highest MVD (183.42 ± 8.24) followed by DLBCL (149.91 ± 13.68). A significant difference was found in MVD between reactive and individual lymphoma groups. SLL had significantly lower MVD than other lymphoma subtypes. *Conclusion*. Angiogenesis, assessed by MVD, showed significant differences among subtypes of NHL, especially the indolent types like SLL. The higher MVD in aggressive lymphomas like PTCL and DLBCL can potentially be utilized in targeted therapy with antiangiogenic drugs.

## 1. Introduction

Angiogenesis or the process of formation of new blood vessels exerts a crucial role in the progression and metastasis of various tumors, including solid organ tumors and hematologic malignancies. Of the various methods to assess angiogenesis, microvessel density (MVD) has been studied extensively in various tumors [1, 2]. Recent studies have shown enhanced angiogenesis in lymphomas, both Hodgkin's disease and non-Hodgkin's lymphoma (NHL) [3, 4]. The results in NHLs have been conflicting in that few studies have demonstrated higher MVD in aggressive subtypes of NHL [4] while others have shown higher MVD in indolent lymphomas [3]. Some authors have also explored association of MVD with angiogenic factors and receptor expression [5]. 

This recent and increasing interest in evaluation of angiogenesis in NHLs arises from the availability of antiangiogenic therapy as a directed therapeutic tool [6]. This mode of therapy has been tried in lymphoma as a single agent or in combination with standard therapeutic protocols with promising results [7]. 

The present study was aimed at assessing the angiogenesis in the form of microvessel density in NHL along with comparison between various subtypes of NHLs, in view of the present conflicting data on this subject. 

## 2. Materials and Methods

This was a retrospective study including 64 consecutive cases of non-Hodgkin's lymphoma (NHL) diagnosed over a period of three years (2008–2010). The patients included 28 females and 36 males, with active disease at stage III or IV. The histological sections were reviewed and NHL subclassified according to the REAL classification system [8]. 

Angiogenesis was assessed using immunohistochemistry for CD34 (Class II, Clone QBEnd 10, DAKO, Denmark) in all the cases. Immunohistochemistry was performed using standard protocols with streptavidin-biotin complex technique and microwave heat-induced antigen retrieval in citrate buffer (pH 6.0). The CD34-stained sections were examined at 100x magnification to delineate “hot spots,” that is, areas of maximal MVD. In three such hot spots in each case, all microvessels (defined as distinct CD34+ cell or cell cluster, irrespective of lumen) were counted at 400x magnification by two investigators independently (each field representing an area of 0.375 mm^2^), and a mean value of both investigators was taken. MVD was calculated and expressed as number of microvessels per mm^2^. For comparison, 15 lymph node biopsies with features of reactive lymphoid hyperplasia were included and concurrently stained with anti-CD34 antibody. MVD was calculated in a similar way in these biopsies as well. 

Statistical test (Student's *t*-test) was applied to assess the significance of difference between various groups. A *P* value of <0.05 was taken as statistically significant.

## 3. Results

**Figure 1:**
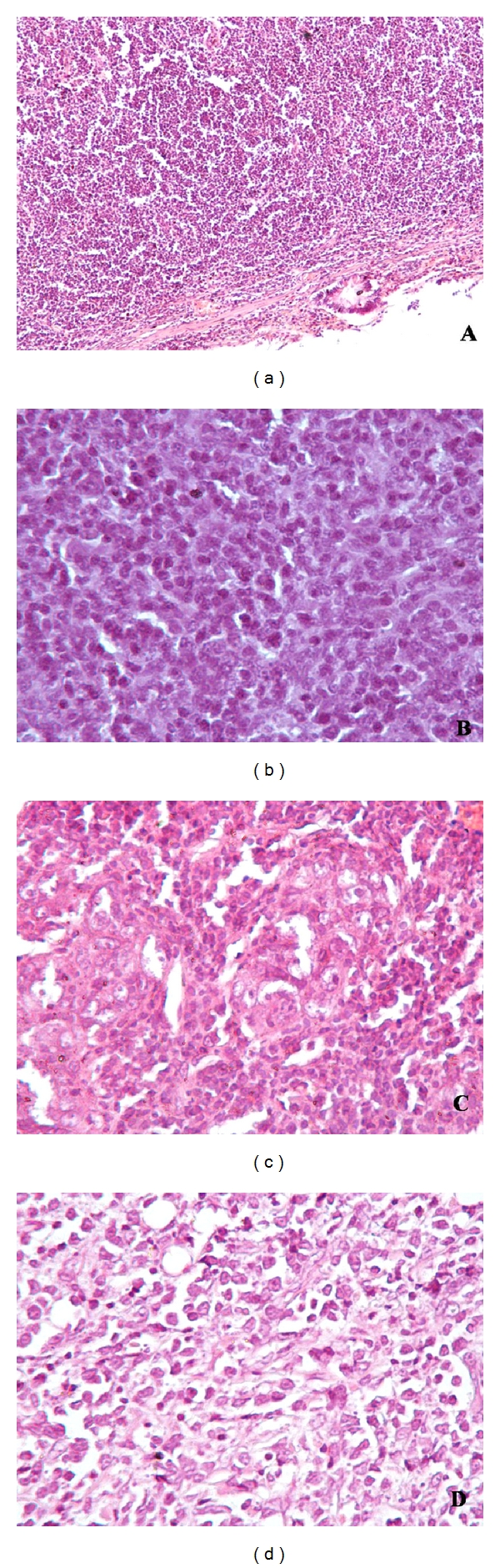
Photomicrographs showing the histomorphology of MALToma ((a) H&E × 100), lymphoblastic lymphoma ((b) H&E × 200), peripheral T-cell lymphoma ((c) H&E × 200), and diffuse large cell lymphoma ((d) H&E × 200).

The subcategorization of the included cases according to the REAL classification is shown in Table 1. Most of the cases were of ML, diffuse large cell type (32.81%) followed by ML, follicular (23.44%) and ML, and lymphoblastic type (15.63%). T-cell NHL comprised 7.81% of our cases (Figure 1).

**Figure 2:**
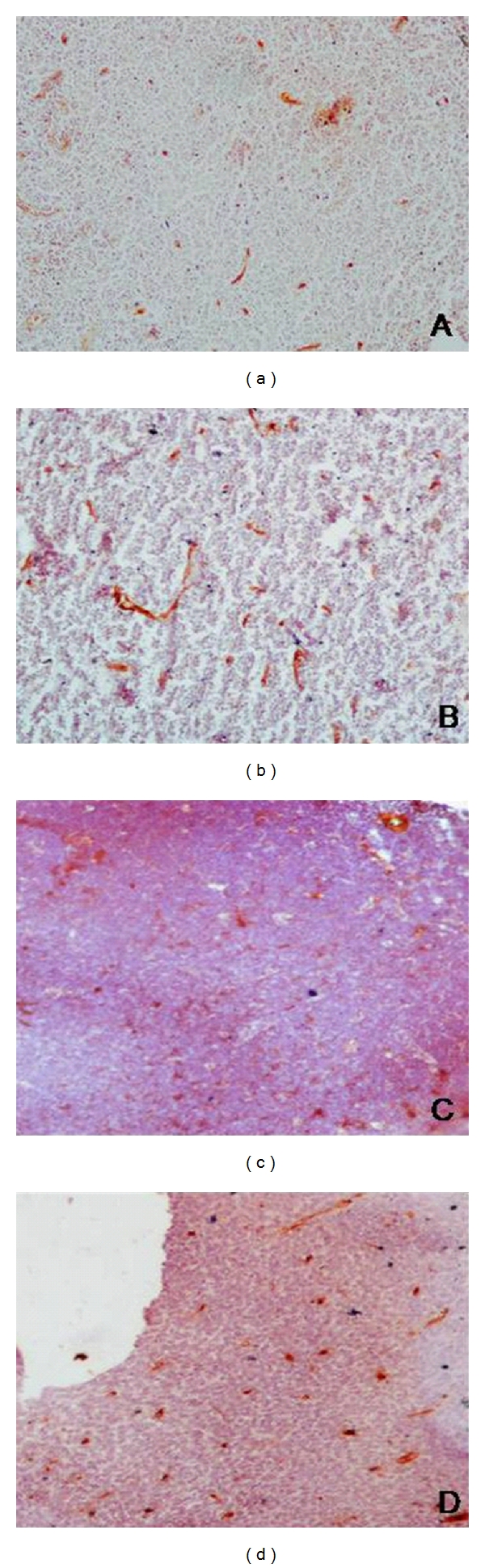
Photomicrographs of immunostained sections from cases of small lymphocytic lymphoma ((a) × 100), MALToma ((b) × 100), lymphoblastic lymphoma ((c) × 100), and diffuse large cell lymphoma ((d) × 100).

**Figure 3:**
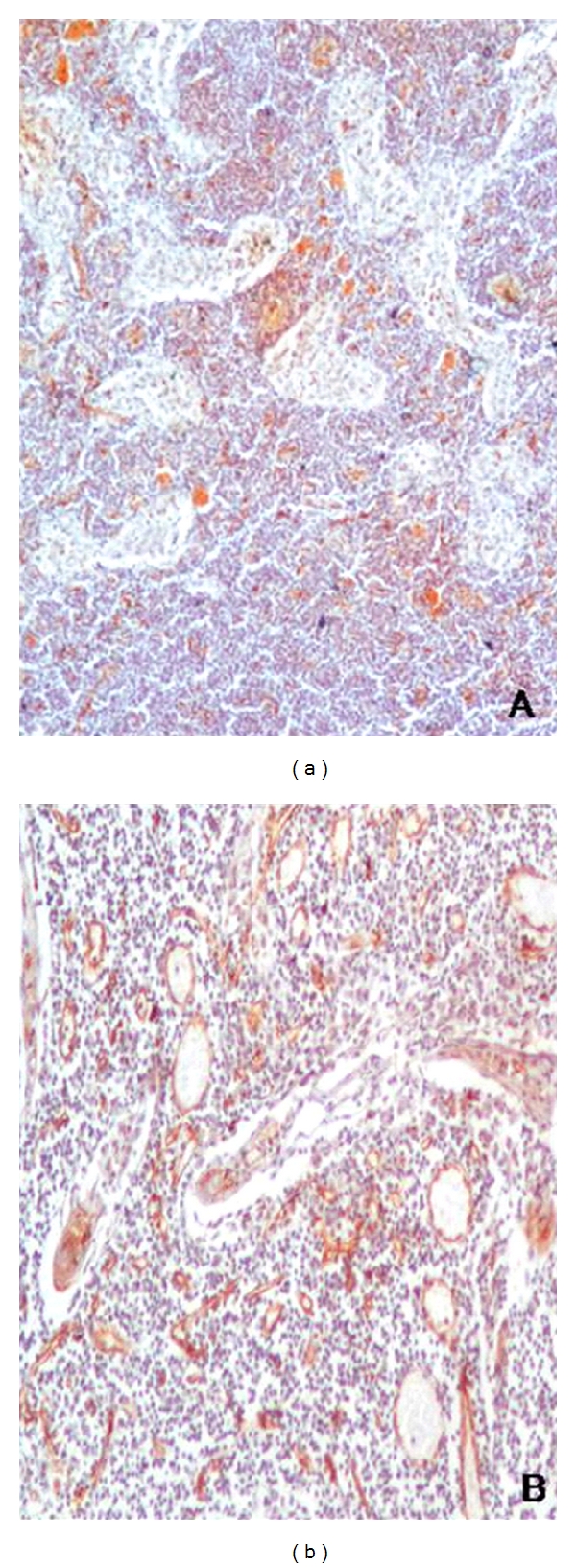
CD34-stained section of a case of reactive lymphoid hyperplasia ((a, b) × 100).

The mean MVD in lymph nodes with reactive lymphoid hyperplasia was 191.92 (±12.16 per mm^2^). This was significantly higher compared to all the lymphomas included in this study. Among lymphomas, the highest MVD was recorded in peripheral T-cell lymphoma (183.42 ± 8.24 per mm^2^) followed by diffuse large B-cell lymphoma (149.91 ± 13.68 per mm^2^) and follicular lymphoma (141.21 ± 23.33 per mm^2^). Small lymphocytic lymphoma had the lowest MVD (76.78 ± 10.41 per mm^2^). The MVD of various groups of NHL and reactive lymphoid hyperplasia is tabulated in Table 2 and Figures 2 and 3.

On statistical analysis (Student's *t*-test), reactive lymphoid hyperplasia showed a significantly higher MVD (*P* < 0.001) than individual NHL subgroups, except PTCL (*P* > 0.05). A significant difference in MVD was also noted between small lymphocytic lymphoma and other NHL categories. PTCL showed a significantly higher MVD (*P* < 0.05) than diffuse large cell lymphoma, follicular lymphoma, lymphoblastic lymphoma, and mucosa-associated lymphoid tissue lymphoma (MALToma). No significant difference in MVD was found between other categories of non-Hodgkin's lymphoma.

## 4. Discussion

Angiogenesis is a multistep process playing a crucial role in progression and metastasis of various tumors, including those of visceral organs and hematolymphoid malignancies. Angiogenesis is required by the tumor for ensuring adequate supply of oxygen and nutrients to the proliferating tumor cell [9]. Angiogenesis can be assessed by several methods, including MVD, microvessel area, angiogenic molecular quantification within the tumor, presence of angiogenic receptors within the tumor, measurement of angiogenic factors within serum, and urine of patients with cancer [1, 2, 10]. Of these methods, MVD has been studied extensively in various tumors.

The NHLs are a diverse group of lymphoproliferative neoplasms with variable clinical behavior and prognosis. Currently, lymphomas are classified on the basis of morphology, immunology, and genetic and clinical features [8]. The clinical stage of the lymphoma is the most important prognostic factor, apart from the histological grade (indolent versus aggressive). In addition, various other features such as age at diagnosis, tumor burden (measured by serum LDH, beta2 microglobulin), tumor proliferation rate, p53 mutations, and T-cell immunophenotype have also been shown to exert prognostic significance in a few studies [11].

Few observational studies in the past have indicated that angiogenesis is enhanced in lymphomas, demonstrated by Wolf and Hubler in a hamster cheek pouch model [12]. Subsequently, other authors demonstrated increased vascularity in Hodgkin's disease and NHL, with higher counts in high-grade lymphomas [3, 4]. Studies exploring angiogenesis by measurement of MVD have shown that MVD scores are the highest in aggressive subtypes including Burkitt's lymphoma and PTCL and the lowest in indolent lymphomas [4]. In contrast, a study evaluating B-cell NHLs showed higher MVD in chronic lymphatic leukemia compared to aggressive mantle cell and diffuse large cell lymphomas [3]. The present study evaluated CD34 staining as marker of tumor vascularity in biopsies with NHL and comparison of various types of NHLs and reactive follicular hyperplasia. The MVD was significantly higher in peripheral T-cell lymphoma and lower in small lymphocytic lymphoma. Our results are in consonance with few of the previous studies [13, 14]. The variability in results of various studies has been ascribed to the range of cell surface markers used, differences in scoring methodology, and heterogeneity of lymphoma stroma [15].

Few earlier studies have correlated MVD with expression of angiogenic factors and receptors. In diffuse large B-cell lymphomas, the association of MVD with tumor cell expression of vascular endothelial growth factor (VEGF) has shown conflicting results in various studies [5, 16]. A recent study found no significant relationship between MVD and patient characteristics like age, gender, stage, nodal status, and response to treatment. The response to therapy was higher in VEGF-negative patients than in VEGF-positive patients [17].

The increasing interest in evaluation of angiogenesis in lymphomas stems from the availability and success of anti-angiogenic therapy in certain solid tumors, like colorectal cancer [6]. Anti-angiogenic drugs have demonstrated modest clinical activity in lymphomas as a single agent and have also been combined with rituximab-CHOP in upfront treatment [7]. The promising result of anti-VEGF therapy has opened an arena of therapeutic possibilities for patients with NHL. The results of the present study suggest enhanced angiogenesis in aggressive lymphomas, like peripheral T-cell lymphomas and diffuse large B-cell lymphomas. On the other hand, indolent lymphomas like small lymphocytic lymphomas and follicular lymphomas had significantly lower MVD. This data can be helpful in assessing the utilization of antiangiogenic therapy in patients with NHL. Further studies are required to confirm these results.

## Figures and Tables

**Table 1:** Classification of patients with NHL included in this study, according to REAL classification.

NHL type	Number of cases	Percentage (%)
ML, small lymphocytic	06	9.38
ML, follicular	15	23.44
ML, diffuse large cell	21	32.81
ML, lymphoblastic	10	15.63
MALTomas	07	10.94
ML, peripheral T cell	05	7.81

ML: malignant lymphoma.

**Table 2:** MVD in various NHL subgroups and reactive lymphoid hyperplasia (expressed as mean micro-vessel count per mm^2^).

NHL type	MVD (per mm^2^) mean ± SD
ML, small lymphocytic	76.78 ± 10.41
ML, follicular	141.21 ± 23.33
ML, diffuse large cell	149.91 ± 13.68
ML, lymphoblastic	124.45 ± 35.94
MALTomas	106.03 ± 32.03
ML, peripheral T cell	183.42 ± 8.24
Reactive lymphoid hyperplasia	191.92 ± 12.16

ML: malignant lymphoma.
